# Supplementary material for: SLC38A10 Deficiency in Mice Affects Plasma Levels of Threonine and Histidine in Males but Not in Females: A Preliminary Characterization Study of SLC38A10−/− Mice
Source: Genes (Basel). 2023 Mar 30;14(4):835. doi: 10.3390/genes14040835 (PMC10138244; doi:10.3390/genes14040835)
Supplement: Supplementary file 1 [file genes-14-00835-s001.zip › Olink data.pdf]

Robert Fredriksson		Olink NPX Manager 0.0.66.0	
NPX data			
Panel		Olink MOUSE EXPLORATORY(v.3801)	
Assay	Costumer ID	Clmp	
Uniprot ID		Q8R373	
OlinkID		OID05027	
75_S0996_ 1:1 WT	5485		6.19739
76_S0997_ 1:1 WT	5556		6.02598
77_S0998_ 1:1 WT	5572		5.80662
78_S0999_ 1:1 KO	5403		5.64804
79_S1000_ 1:1 KO	5568		5.28006
80_S1001_ 1:1 KO	5569		5.70499
LOD			0.39859
Missing Data freq.			0%
		wt	6.01000
		ko	5.54436
		diff.	-0.46563

Olink MOUSE EXPLORATORY(v.3801)	Olink MOUSE EXPLORATORY(v.3801)
Matn2	Cpe
O08746	Q00493
OID05028	OID05029
3.62818	5.88031
3.68956	5.80532
3.64758	5.59125
2.92600	5.51049
2.90979	5.46797
3.66552	5.95750
0.94183	0.59951
0%	0%
3.65511	5.75896
3.16710	5.64532
-0.48800	-0.11364

Olink MOUSE EXPLORATORY(v.3801)	Olink MOUSE EXPLORATORY(v.3801)
Gcg	Yes1
P55095	Q04736
OID05031	OID05033
2.53023	0.83292
2.54180	1.65405
1.44458	1.21094
1.47602	0.62223
2.30376	0.88127
4.73617	1.41091
0.84528	0.57656
0%	0%
2.17220	1.23264
2.83865	0.97147
0.66645	-0.26117

Olink MOUSE EXPLORATORY(v.3801)	Olink MOUSE EXPLORATORY(v.3801)
Il17a	Foxo1
Q62386	Q9R1E0
OID05034	OID05035
3.30968	0.67022
2.64306	1.59891
2.50181	0.92899
3.46181	1.43616
2.10490	0.71273
2.86630	1.05559
0.56770	0.44248
0%	0%
2.81818	1.06604
2.81100	1.06816
-0.00718	0.00212

Olink MOUSE EXPLORATORY(v.3801)	Olink MOUSE EXPLORATORY(v.3801)
Tnfrsf11b	Tgfb1
O08712	P04202
OID05036	OID05037
4.45952	2.04832
4.59570	1.67505
4.27416	1.90857
4.10297	1.67593
3.88533	1.54930
4.58690	1.97904
0.47326	0.48002
0%	0%
4.44313	1.87731
4.19173	1.73476
-0.25139	-0.14256

Olink MOUSE EXPLORATORY(v.3801)	Olink MOUSE EXPLORATORY(v.3801)
Pla2g4a	Prdx5
P47713	P99029
OID05038	OID05040
6.79788	3.39797
7.03765	4.83719
6.73270	3.47133
6.50781	2.94081
6.00126	3.35681
7.18812	4.33643
0.50735	0.94595
0%	0%
6.85608	3.90216
6.56573	3.54468
-0.29035	-0.35748

Olink MOUSE EXPLORATORY(v.3801)	Olink MOUSE EXPLORATORY(v.3801)
Tgfa	Ccl5
P48030	P30882
OID05041	OID05042
4.71989	1.17742
5.24685	1.09683
5.11204	0.86568
4.33790	1.19849
4.65078	1.01813
5.96676	0.89825
0.71431	0.59622
0%	0%
5.02626	1.04664
4.98515	1.03829
-0.04111	-0.00835

Olink MOUSE EXPLORATORY(v.3801)	Olink MOUSE EXPLORATORY(v.3801)
Epo	Axin1
P07321	O35625
OID05043	OID05044
1.36056	2.69324
1.61988	3.49558
2.42608	2.79034
1.04122	2.98451
1.59604	2.93531
2.68634	3.52225
0.50793	0.69934
0%	0%
1.80217	2.99305
1.77453	3.14736
-0.02764	0.15430

Olink MOUSE EXPLORATORY(v.3801)	Olink MOUSE EXPLORATORY(v.3801)
Fst	Rgma
P47931	Q6PCX7
OID05046	OID05047
7.79273	1.99248
7.95448	1.96647
8.45659	1.66175
7.68250	1.52105
7.88733	1.48741
8.10494	1.59773
0.60319	0.66254
0%	0%
8.06793	1.87357
7.89159	1.53540
-0.17634	-0.33817

Olink MOUSE EXPLORATORY(v.3801)	Olink MOUSE EXPLORATORY(v.3801)
Nadk	Tnni3
P58058	P48787
OID05048	OID05049
5.36104	10.71685
7.13591	8.92410
6.39673	9.01444
6.43435	7.83804
6.09833	8.66594
7.15615	10.62843
0.43084	0.76967
0%	0%
6.29789	9.55180
6.56294	9.04414
0.26505	-0.50766

Olink MOUSE EXPLORATORY(v.3801)	Olink MOUSE EXPLORATORY(v.3801)
Notch3	Snap29
Q61982	Q9ERB0
OID05050	OID05051
4.18064	6.42906
4.05110	7.30916
4.05893	6.83404
4.02424	6.94794
4.25723	6.80255
3.94114	7.52228
0.84463	0.68922
0%	0%
4.09689	6.85742
4.07420	7.09092
-0.02269	0.23350

Olink MOUSE EXPLORATORY(v.3801)	Olink MOUSE EXPLORATORY(v.3801)
Cntn1	Clstn2
P12960	Q9ER65
OID05052	OID05053
5.25998	1.44885
5.05918	1.65053
4.97873	1.11097
4.94272	0.88610
4.79125	1.14625
5.10982	1.09070
0.44920	0.87581
0%	0%
5.09930	1.40345
4.94793	1.04102
-0.15137	-0.36243

Olink MOUSE EXPLORATORY(v.3801)	Olink MOUSE EXPLORATORY(v.3801)
S100a4	Ca13
P07091	Q9D6N1
OID05054	OID05055
4.83796	1.23393
6.12113	3.45374
5.56145	1.86493
4.04025	1.21395
4.14913	1.86103
5.30106	2.70490
0.95806	0.88355
0%	0%
5.50685	2.18420
4.49681	1.92663
-1.01003	-0.25757

Olink MOUSE EXPLORATORY(v.3801)	Olink MOUSE EXPLORATORY(v.3801)
Mia	Cant1
Q61865	Q8VCF1
OID05056	OID05057
4.19571	1.06905
4.35737	1.42670
3.79109	1.15269
3.80603	1.20258
3.63868	1.02931
3.87326	1.14972
0.35726	0.99015
0%	0%
4.11472	1.21615
3.77266	1.12720
-0.34207	-0.08894

Olink MOUSE EXPLORATORY(v.3801)	Olink MOUSE EXPLORATORY(v.3801)
Gfra1	Ppp1r2
P97785	Q9DCL8
OID05059	OID05060
2.55604	1.92815
2.87738	3.09679
2.49928	2.39647
1.99546	2.02244
2.38847	2.03602
2.27230	2.59791
0.83604	0.98570
8%	0%
2.64423	2.47380
2.21874	2.21879
-0.42549	-0.25501

Olink MOUSE EXPLORATORY(v.3801)	Olink MOUSE EXPLORATORY(v.3801)
Adam23	Cyr61
Q9R1V7	P18406
OID05061	OID05063
1.75140	4.11722
1.71245	3.40768
1.43216	4.46286
1.05106	4.52882
0.99244	3.61514
1.53661	4.80880
0.38353	0.68970
0%	0%
1.63200	3.99592
1.19337	4.31759
-0.43863	0.32167

Olink MOUSE EXPLORATORY(v.3801)	Olink MOUSE EXPLORATORY(v.3801)
Dlk1	Ahr
Q09163	P30561
OID05064	OID05065
2.47371	1.04700
2.13658	3.47298
1.89973	1.54650
1.66946	1.14756
1.43687	1.36976
1.76600	2.28303
0.69723	0.79212
0%	0%
2.17001	2.02216
1.62411	1.60012
-0.54590	-0.42204

Olink MOUSE EXPLORATORY(v.3801)	Olink MOUSE EXPLORATORY(v.3801)
Ccl2	Eno2
P10148	P17183
OID05066	OID05067
8.93194	1.05961
8.86260	1.27662
10.64850	1.37743
9.15060	1.34416
8.55190	1.12847
11.17937	1.47535
0.54657	0.65349
0%	0%
9.48101	1.23789
9.62729	1.31599
0.14628	0.07811

Olink MOUSE EXPLORATORY(v.3801)	Olink MOUSE EXPLORATORY(v.3801)
Wfikkn2	Flrt2
Q7TQN3	Q8BLU0
OID05069	OID05070
4.12512	3.64474
3.84674	3.77208
3.91026	3.01656
3.69199	3.00055
3.53874	2.92023
3.92311	3.39779
0.43479	0.51849
0%	0%
3.96071	3.47779
3.71795	3.10619
-0.24276	-0.37160

Olink MOUSE EXPLORATORY(v.3801)	Olink MOUSE EXPLORATORY(v.3801)
Qdpr	Fas
Q8BVI4	P25446
OID05071	OID05074
4.16911	3.08644
4.86961	3.28979
4.80615	3.07486
4.37806	3.07170
4.52261	2.79638
4.93401	3.34567
0.80171	0.63539
0%	0%
4.61496	3.15036
4.61156	3.07125
-0.00340	-0.07911

Olink MOUSE EXPLORATORY(v.3801)	Olink MOUSE EXPLORATORY(v.3801)
Erbb4	Riox2
Q61527	Q8CD15
OID05075	OID05076
3.41161	3.22531
3.09813	4.26255
3.21663	2.97482
3.13553	4.51701
3.16873	3.62056
3.06350	3.64711
0.77040	0.84677
0%	0%
3.24212	3.48756
3.12259	3.92823
-0.11954	0.44067

Olink MOUSE EXPLORATORY(v.3801)	Olink MOUSE EXPLORATORY(v.3801)
Plxna4	Epcam
Q80UG2	Q99JW5
OID05077	OID05078
2.75877	2.37962
2.83176	2.17693
2.18025	2.16524
1.74687	2.09788
1.37559	1.95560
1.93157	2.38389
0.78240	0.62309
0%	0%
2.59026	2.24060
1.68468	2.14579
-0.90558	-0.09481

Olink MOUSE EXPLORATORY(v.3801)	Olink MOUSE EXPLORATORY(v.3801)
Ccl3	Vsig2
P10855	Q9Z109
OID05079	OID05081
2.54797	2.83418
2.95880	2.54989
2.34216	2.20374
2.84568	2.55290
2.03881	1.65824
2.48885	3.24914
0.41778	0.79201
0%	0%
2.61631	2.52927
2.45778	2.48676
-0.15853	-0.04251

Olink MOUSE EXPLORATORY(v.3801)	Olink MOUSE EXPLORATORY(v.3801)
Hgf	Sez6l2
Q08048	Q4V9Z5
OID05082	OID05083
4.20178	3.75841
4.07645	3.60806
4.18790	3.63619
4.12569	3.54426
3.59756	3.39556
4.57544	3.81008
0.50936	0.44953
0%	0%
4.15538	3.66755
4.09956	3.58330
-0.05581	-0.08425

Olink MOUSE EXPLORATORY(v.3801)	Olink MOUSE EXPLORATORY(v.3801)
Il1a	Il23r
P01582	Q8K4B4
OID05084	OID05085
3.99903	4.51829
5.05778	4.45192
4.75270	4.37039
4.07375	4.16980
3.56366	4.12002
5.70881	4.20957
1.05211	0.69518
0%	0%
4.60317	4.44687
4.44874	4.16646
-0.15443	-0.28040

Olink MOUSE EXPLORATORY(v.3801)	Olink MOUSE EXPLORATORY(v.3801)
Dll1	Tnfrsf12a
Q61483	Q9CR75
OID05086	OID05089
4.15615	3.95278
3.96518	4.07565
4.03778	3.88636
3.73341	3.81746
3.67489	3.51256
3.87365	4.32675
0.61304	0.63562
0%	0%
4.05304	3.97160
3.76065	3.88559
-0.29239	-0.08601

Olink MOUSE EXPLORATORY(v.3801)	Olink MOUSE EXPLORATORY(v.3801)
Acvrl1	Lgmn
Q61288	O89017
OID05090	OID05091
3.37463	4.67804
3.51979	4.61140
3.60212	4.47008
3.13378	4.40670
2.56637	4.31638
3.53427	5.05111
0.67510	0.39083
0%	0%
3.49885	4.58651
3.07814	4.59140
-0.42071	0.00489

Olink MOUSE EXPLORATORY(v.3801)	Olink MOUSE EXPLORATORY(v.3801)
Cxcl9	Map2k6
P18340	P70236
OID05093	OID05094
4.43315	5.54627
4.49533	6.13673
4.18245	5.75862
4.42911	5.58719
4.03715	5.35792
4.77840	6.22606
1.09895	0.77335
0%	0%
4.37031	5.81387
4.41489	5.72372
0.04458	-0.09015

Olink MOUSE EXPLORATORY(v.3801)	Olink MOUSE EXPLORATORY(v.3801)
Il17f	Casp3
Q7TNI7	P70677
OID05096	OID05098
1.48262	6.67579
1.85421	7.53911
1.29901	7.28954
1.96751	7.07703
0.98751	7.02317
1.35690	7.51915
0.38120	0.99185
8%	0%
1.54528	7.16815
1.43731	7.20645
-0.10797	0.03830

Olink MOUSE EXPLORATORY(v.3801)	Olink MOUSE EXPLORATORY(v.3801)
Apbb1ip	Wisp1
Q8R5A3	O54775
OID05099	OID05100
2.35049	4.44495
4.11986	4.48662
2.90129	4.06671
2.28201	3.68931
2.41933	3.42955
3.15757	4.00593
0.58266	0.63232
0%	0%
3.12388	4.33276
2.61964	3.70826
-0.50424	-0.62450

Olink MOUSE EXPLORATORY(v.3801)	Olink MOUSE EXPLORATORY(v.3801)
Cdh6	Pdgfb
P97326	P31240
OID05101	OID05102
3.47805	7.17340
3.10258	6.70555
2.64686	6.96102
2.57514	7.17584
2.44818	6.24611
2.91201	8.00535
0.52073	0.76255
0%	0%
3.07583	6.94666
2.64511	7.14243
-0.43072	0.19578

Olink MOUSE EXPLORATORY(v.3801)	Olink MOUSE EXPLORATORY(v.3801)
Igsf3	Tgfbr3
Q6ZQA6	O88393
OID05103	OID05104
3.71066	3.33638
3.69881	3.25351
3.38217	2.90751
3.20425	2.97730
2.89557	2.69976
3.43510	3.15967
0.75693	0.67348
0%	0%
3.59721	3.16580
3.17831	2.94558
-0.41891	-0.22022

Olink MOUSE EXPLORATORY(v.3801)	Olink MOUSE EXPLORATORY(v.3801)
Cxcl1	Cntn4
P12850	Q69Z26
OID05105	OID05107
7.17493	3.15504
6.64749	3.07575
8.24766	2.70432
7.71391	2.63478
7.06536	2.55936
9.53995	2.68055
0.75853	0.52251
0%	0%
7.35669	2.97837
8.10641	2.62490
0.74971	-0.35347

Olink MOUSE EXPLORATORY(v.3801)	Olink MOUSE EXPLORATORY(v.3801)
Ghrl	Lpl
Q9EQX0	P11152
OID05108	OID05109
4.14064	2.54311
3.33490	3.16795
3.59461	3.10071
3.76212	2.55333
3.74417	2.51726
3.89681	2.96515
0.73249	0.78778
0%	0%
3.69005	2.93726
3.80103	2.67858
0.11098	-0.25868

Olink MOUSE EXPLORATORY(v.3801)	Olink MOUSE EXPLORATORY(v.3801)
Fstl3	Dctn2
Q9EQC7	Q99KJ8
OID05110	OID05111
4.88298	1.41590
5.10766	2.04747
5.11334	1.80969
4.69641	1.09446
4.70435	1.28512
5.11297	2.45523
0.59024	0.65914
0%	0%
5.03466	1.75769
4.83791	1.61160
-0.19675	-0.14608

Olink MOUSE EXPLORATORY(v.3801)	Olink MOUSE EXPLORATORY(v.3801)
Eda2r	Ntf3
Q8BX35	P20181
OID05113	OID05114
5.13658	1.14200
5.57395	1.36382
4.46939	1.30706
5.19546	1.18115
4.44056	0.79930
5.48957	1.26653
0.59127	0.53937
0%	0%
5.05997	1.27096
5.04186	1.08233
-0.01811	-0.18863

Olink MOUSE EXPLORATORY(v.3801)	Olink MOUSE EXPLORATORY(v.3801)
Tnfsf12	Ccl20
O54907	O89093
OID05115	OID05116
3.13326	7.91108
3.39144	7.70904
3.00340	7.68905
3.26941	7.96773
2.62483	9.93077
3.23257	10.93554
1.03385	0.94510
0%	0%
3.17603	7.76972
3.04227	9.61135
-0.13376	1.84162

Olink MOUSE EXPLORATORY(v.3801)	Olink MOUSE EXPLORATORY(v.3801)
Fli1	Tpp1
P26323	O89023
OID05117	OID05118
1.82881	5.66482
3.51842	5.82406
1.94251	5.45497
2.81458	5.50821
2.21755	5.43209
2.14641	6.01584
0.71776	0.51503
0%	0%
2.42991	5.64795
2.39285	5.65205
-0.03707	0.00410

Olink MOUSE EXPLORATORY(v.3801)	Olink MOUSE EXPLORATORY(v.3801)
Tnr	Vegfd
Q8BYI9	P97946
OID05119	OID05120
2.81144	2.61269
2.69629	2.54097
2.99199	2.36177
2.41745	2.20144
2.38387	1.99774
2.44973	2.68741
0.76322	0.66651
0%	0%
2.83324	2.50514
2.41702	2.29553
-0.41622	-0.20961

Olink MOUSE EXPLORATORY(v.3801)<br>Parp1<br>P11103<br>OID05121	Olink MOUSE EXPLORATORY<br>Plate ID	Olink MOUSE EXPLORATORY<br>QC Warning
6.71445	Heatmap #2.csv	Pass
8.50868	Heatmap #2.csv	Pass
6.51134	Heatmap #2.csv	Pass
8.23095	Heatmap #2.csv	Pass
7.21255	Heatmap #2.csv	Pass
6.75798	Heatmap #2.csv	Pass
1.01162		
0%		
7.24482		
7.40049		
0.15567		
