# Supplementary material for: SLC38A10 Deficiency in Mice Affects Plasma Levels of Threonine and Histidine in Males but Not in Females: A Preliminary Characterization Study of SLC38A10−/− Mice
Source: Genes (Basel). 2023 Mar 30;14(4):835. doi: 10.3390/genes14040835 (PMC10138244; doi:10.3390/genes14040835)
Supplement: Supplementary file 1 [file genes-14-00835-s001.zip › Supplementary_Material.pdf]

Supplementary Material

Table S1. Amino acid measurement by LC-MSMS. Retention times (rt), MRM-transition stages monitored (precursor ion and product ions) and collision energies of analyzed compounds.

Compounds	MRM transition		rt (min)	Collision Energy (V)
	Precursor Ion	Product Ion		
alanine	260.1	171	4.1	14
arginine	345.1	171	2.8	35
aspartic acid	304.1	171	3.4	18
cystine	581	171	5.2	25
glutamic acid	318.1	171	3.6	22
glycine	246.1	171	3.1	18
histidine	326.1	171	2.6	26
isoleucine	302.1	171	7.8	18
leucine	302.1	171	7.6	18
lysine	487.2	171	5.1	26
methionine	320.1	171	6.0	22
phenylalanine	336.1	171	8.1	18
proline	286.1	171	4.4	14

serine	276.1	171	3.1	14
threonine	290.1	171	3.7	18
tyrosine	352.1	171	5.7	18
valine	288.1	171	6.1	18
citrulline	346.2	171	3.4	30
GABA	274.1	171	4.1	18
glutamine	317.1	171	3.0	22
asparagine	303.1	171	2.8	18
ornithine	473.2	171	4.7	34
tryptophan	375.2	171	8.4	26
kynurenine	379.2	171	7.6	37
Labelled internal standards				
alanine (^{13}C3, ^{15}N)	264.07	171	4.1	14
arginine ($^{13}C_6$, $^{15}N_4$)	355.1	171	2.8	35
aspartic acid ($^{13}C_4$, ^{15}N)	309.07	171	3.3	18
cystine ($^{13}C_6$, $^{15}N_2$)	589	171	5.2	25
glutamic acid ($^{13}C_5$, ^{15}N)	324.09	171	3.5	22
glycine ($^{13}C_2$, ^{15}N)	249.05	171	3.1	18
histidine ($^{13}C_6$, $^{15}N_3$)	335	171	2.6	26

isoleucine ($^{13}C_6$, ^{15}N)	309.12	171	7.9	18
leucine ($^{13}C_6$, ^{15}N)	309.12	171	7.6	18
lysine ($^{13}C_6$, $^{15}N_2$)	495.1	171	5.2	26
methionine ($^{13}C_5$, ^{15}N)	326.17	171	6.0	22
phenylalanine ($^{13}C_9$, ^{15}N)	346	171	8.2	18
proline ($^{13}C_5$, ^{15}N)	292.09	171	4.4	14
serine ($^{13}C_3$, ^{15}N)	280.6	171	3.0	14
threonine ($^{13}C_4$, ^{15}N)	295.08	171	3.7	18
tyrosine ($^{13}C_9$, ^{15}N)	362.12	171	5.7	18
valine ($^{13}C_5$, ^{15}N)	294.1	171	6.1	18
citrulline (d4)	350.1	171	3.4	30
GABA ($^{13}C_4$)	278.1	171	4.0	18
glutamine ($^{13}C_5$)	322.1	171	3.1	22
asparagine($^{13}C_4$)	307.1	171	2.8	18
ornithine (d6)	479	171	4.6	34
tryptophan (d8)	383.2	171	8.3	26
kynurenine (d4)	383	171	7.7	37

A

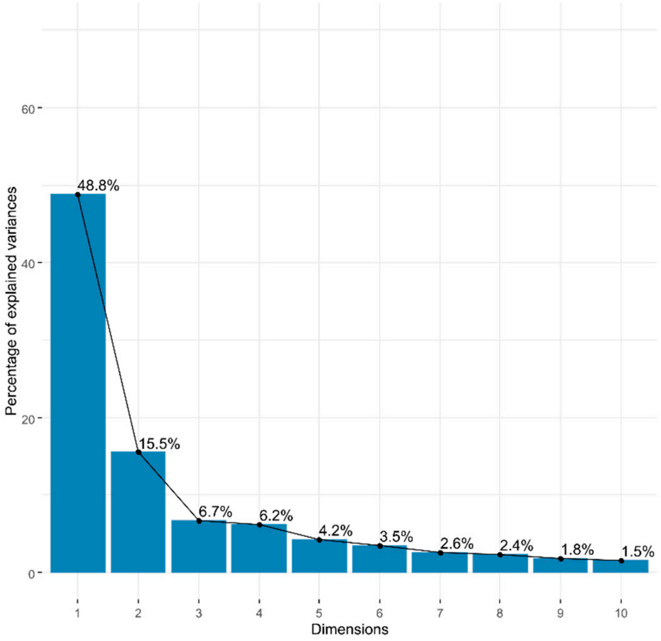

B

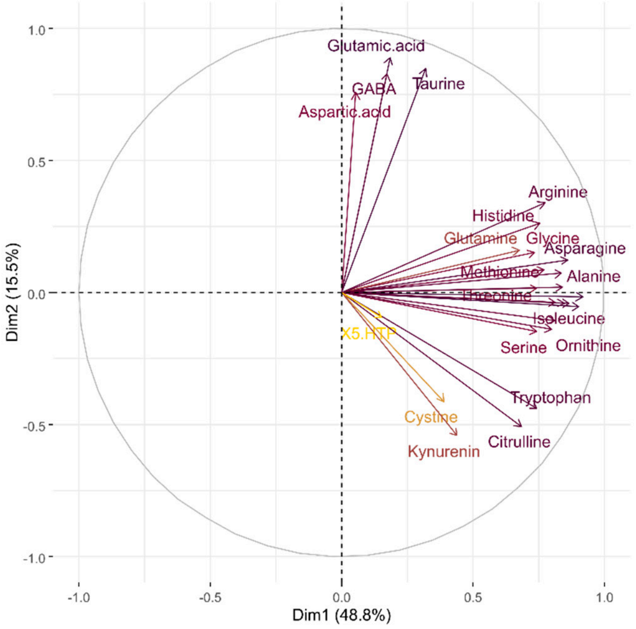

Figure S1. Scree and loading plot results from the principal component analysis (PCA) of amino acid levels in plasma in SLC38A10 deficient mice and their littermate controls. (A) The scree plot shows the components in the PCA, and how much each component explains the variation in the data. The first two dimensions together explain 64.3 % of the variance in the data set. (**B**) The

loading plot showing the variables that impacted the PCA most. The furthest away from the origo, the more the variable impacted on the analysis.

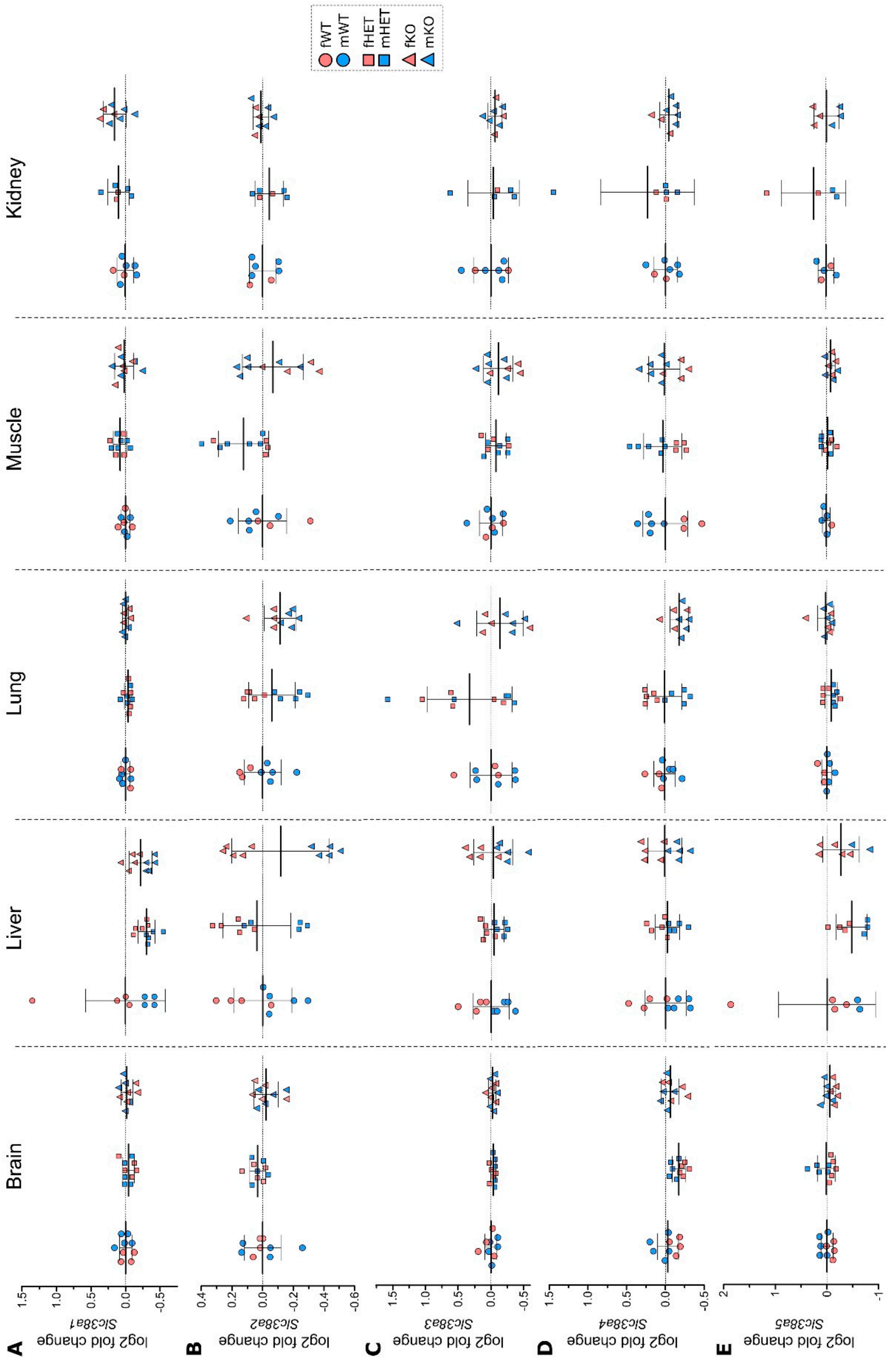

Figure S2. Expression analysis in brain, liver, lung, muscle, and kidney of SLC38A10 deficient mice. RT-qPCR was used to examine the relative expression pattern of (**A**) *Slc38a1*, (**B**) *Slc38a2*, (**C**) *Slc38a3*, (**D**) *Slc38a4* and (**E**) *Slc38a5* in WT, HET and KO mice. Relative expression is shown as log2 fold changes with WT group set as control. Analysis was made with one-way ANOVA with correction for multiple testing (according to qbase+ software version 3.2, Biogazelle, Zwijnaarde, Belgium -www.qbase.com). Data were analyzed with sex collapsed, but data points are colored according to the sex of the animal (males in blue; females in dark pink).

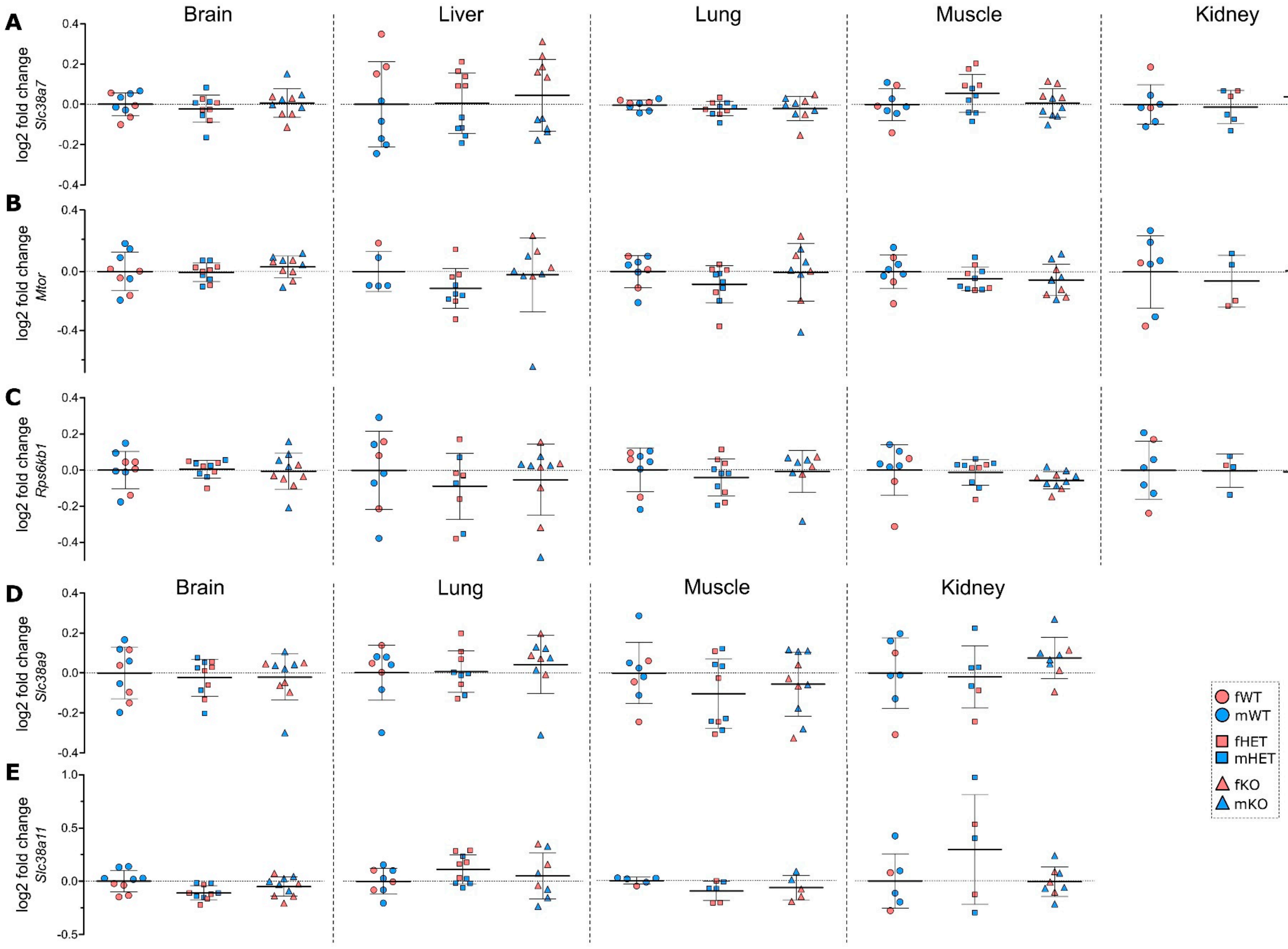

A
B
C
D
E
Brain
Liver
Lung
Muscle
Kidney
log2 fold change Slc38a7
log2 fold change Mtor
log2 fold change Rps6kb1
log2 fold change Slc38a9
log2 fold change Slc38a11
0.4
0.2
0.0
-0.2
-0.4
1.0
0.5
-0.5
fWT
mWT
fHET
mHET
fKO
mKO

Figure S3. Expression analysis in brain, liver, lung, muscle and kidney of SLC38A10 deficient mice. RT-qPCR was used to examine the relative expression pattern of (**A**) *Slc38a7*, (**B**) *Mtor*, (**C**) *Rps6kb1*, (**D**) *Slc38a9* and (**E**) *Slc38a11* in WT, HET and KO mice. Relative expression is shown as log2 fold changes with WT group set as control. Analysis was made with one-way ANOVA with correction for multiple testing (according to qbase+ software version 3.2, Biogazelle, Zwijnaarde, Belgium -www.qbase.com). Data were analyzed with sex collapsed, but data points are colored according to the sex of the animal (males in blue; females in dark pink).

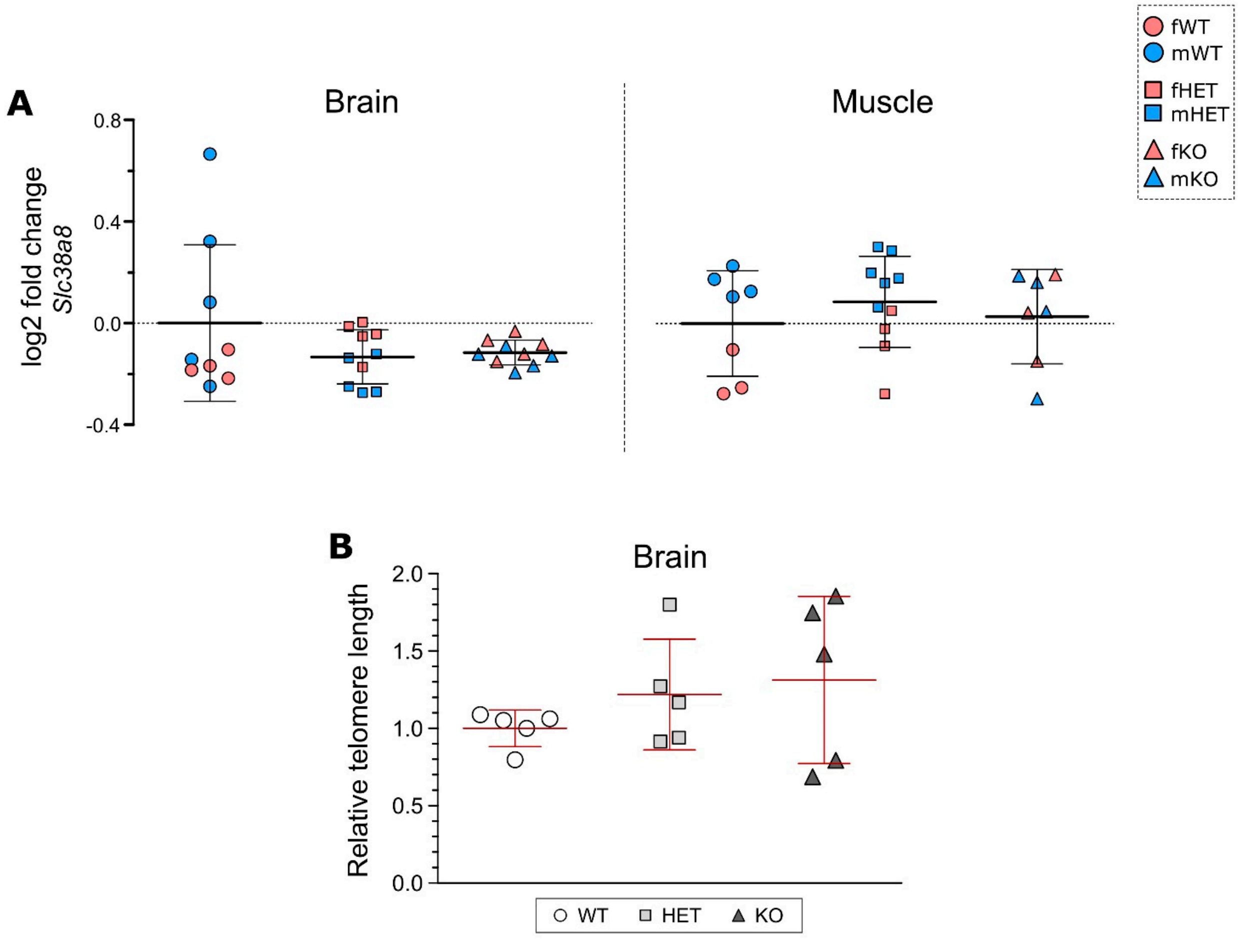

Figure S4. Expression analysis in brain and muscle, as well as a relative telomere length measurement in brain of SLC38A10 deficient mice. (**A**) RT-qPCR was used to examine the relative expression pattern of *Slc38a8* in brain and muscle. Relative expression is shown as log2 fold changes with WT group set as control. Analysis was made with one-way ANOVA with correction for multiple testing (according to qbase+ software version 3.2, Biogazelle, Zwijnaarde, Belgium - www.qbase.com). Data were analyzed with sex collapsed, but data points are colored according to the sex of the animal (males in blue; females in dark pink) (**B**) Genomic DNA from cerebellum and

cortex of ten-week-old males were used for relative telomere measurement. Data were analyzed with a one-way ANOVA with Bonferroni correction. Data is presented as mean values with standard deviation.

Table S2. Protein concentration and number of identified proteins in respective sample from SLC38A10 deficient mice and WT control. Brains from seven week old male mice (n=5/genotype, only WT and KO males used) were dissected and used for protein measurement by LC-MSMS (SciLifeLab, Mass Spectrometry Facility, Uppsala, Sweden).

Sample	Protein concentration (µg/µL)	No. identified proteins
KO1	4,3	2127
KO2	4,6	2166
KO3	4,9	2227
KO4	4,6	2109
KO5	4,9	2116
WT1	4,4	2140
WT2	5,1	2106
WT3	5,0	2248
WT4	4,6	2270
WT5	4,2	2020

Table S3. Proteins significantly regulated in KO brains compared to WT (one missing value). Brains from seven week old male mice (n=5/genotype, only WT and KO males used) were dissected and used for protein measurement by LC-MSMS (SciLifeLab, Mass Spectrometry Facility, Uppsala, Sweden). However, no proteins detected fulfilled the cut-off ratio criteria (two-fold change for upregulation, 0.5 for downregulation).

Protein name	Gene name	Ratio	p-value
**Down-regulated proteins**			
Glycerol-3-phosphate dehydrogenase, mitochondrial	Gpd2	0,94	0,011
Disks large homolog 1	Dlg1	0,94	0,012
Aldehyde dehydrogenase, mitochondrial	Aldh2	0,94	0,026
40S ribosomal protein S14	rps14;Rps14	0,93	0,049
Vesicle-associated membrane protein-associated protein A	Vapa	0,92	0,021
Phosphatidylinositol 4-phosphate 5-kinase type-1 gamma	Pip5k1c	0,92	0,048
78 kDa glucose-regulated protein	Hspa5	0,91	0,009

Phospholipase A-2-activating protein	Plaa	0,90	0,015
Nascent polypeptide-associated complex subunit alpha	Naca	0,90	0,015
60S ribosomal protein L17	Rpl17	0,90	0,003
Proteasome subunit beta type-4	Psmb4	0,90	0,023
Fermitin family homolog 2	Fermt2	0,90	0,048
IQ motif and SEC7 domain-containing protein 1	Iqsec1	0,90	0,043
ATP synthase-coupling factor 6, mitochondrial	Atp5j	0,90	0,018
ADP-ribosylation factor GTPase-activating protein 1	Arfgap1	0,90	0,010
Neurobeachin	Nbea	0,89	0,041
UTP-glucose-1-phosphate uridylyltransferase	Ugp2	0,88	0,010
Myosin-9	Myh9	0,88	0,003
Lipid phosphate phosphohydrolase 3	Ppap2b	0,86	0,016
Prelamin-A/C;Lamin-A/C	Lmna	0,86	0,022
Acyl-CoA synthetase family member 2, mitochondrial	Acsf2	0,84	0,014
Methyl-CpG-binding protein 2	Mecp2	0,84	0,046
Heterogeneous nuclear ribonucleoprotein A/B	Hnrnpab	0,83	0,037
Microtubule-associated protein RP/EB family member 1	Mapre1	0,83	0,027
U1 small nuclear ribonucleoprotein 70 kDa	Snrnp70	0,83	0,028
Coronin-2B; Coronin	Coro2b	0,82	0,008
40S ribosomal protein S20	Rps20	0,82	0,042
Four and a half LIM domains protein 1	Fhl1	0,82	0,014
Glutamate receptor 1	Gria1	0,81	0,027
Charged multivesicular body protein 5	Chmp5	0,80	0,009
Large proline-rich protein BAG6	Bag6	0,80	0,039

Epsin-2	Epn2	0,80	0,023
Serine/arginine-rich splicing factor 7	Srsf7	0,78	0,011
Density-regulated protein	Denr	0,78	0,014
Non-POU domain-containing octamer-binding protein	Nono	0,77	0,038
Talin-1	Tln1	0,73	0,012
Glycerophosphodiester phosphodiesterase domain-containing protein 1	Gdpd1	0,72	0,045
**Up-regulated proteins**			
Thioredoxin-related transmembrane protein 4	Tmx4	1,26	0,005
Ciliary neurotrophic factor receptor subunit alpha	Cntfr	1,26	0,001
Sulfotransferase 4A1;Sulfotransferase	Sult4a1	1,22	0,013
Glia maturation factor beta	Gmfb	1,19	0,014
Glucose 1,6-bisphosphate synthase	Pgm2l1	1,18	0,003
Protein phosphatase 1F	Ppm1f	1,18	0,024
GTP-binding protein Di-Ras1	Diras1	1,17	0,031
Signal transducing adapter molecule 1	Stam	1,17	0,030
Plastin-3	Pls3	1,16	0,013
Putative uncharacterized protein	Skp1a	1,16	0,009
Serine/threonine-protein phosphatase 2A activator	Ppp2r4	1,11	0,043
Phenylalanine--tRNA ligase beta subunit	Farsb	1,10	0,026
Protein NipSnap homolog 1	Nipsnap1	1,10	0,044
Mitochondrial import inner membrane translocase subunit TIM50	Timm50	1,10	0,034
Adenylosuccinate synthetase isozyme 2	Adss	1,09	0,046
Arf-GAP with SH3 domain, ANK repeat and PH domain-containing protein 1	Asap1	1,06	0,036
